# Neutrophil-Lymphocyte Ratio in Patients With Acute Schizophrenia

**DOI:** 10.7759/cureus.52181

**Published:** 2024-01-12

**Authors:** Shutaro Sugita, Hiroi Tomioka, Kensuke Mera, Taro Tazaki, Hana Nishiyama, Hiroki Yamada, Kenji Sanada, Atsuko Inamoto, Akira Iwanami

**Affiliations:** 1 Department of Psychiatry, Showa University School of Medicine, Tokyo, JPN; 2 Mental Care Center, Showa University Northern Yokohama Hospital, Kanagawa, JPN

**Keywords:** neutrophil-lymphocyte ratio, neutrophil-albumin ratio, psychomotor excitement, inflammation, schizophrenia

## Abstract

Introduction

Schizophrenia symptom severity is linked to neuroinflammation. Certain blood cell indexes such as neutrophil-lymphocyte ratio (NLR) and neutrophil-albumin ratio (NAR) have been used as biomarkers in various diseases, including schizophrenia. In acute clinical practice, it is challenging to decide whether to provide intravenous antipsychotic treatment in some cases due to the lack of objective biomarkers of psychiatric symptoms. The NLR of individuals with schizophrenia is thought to be associated with disease severity, and changes in NLR may reflect a patient's response to antipsychotic treatment. We investigated the application of NLR as a biomarker for identifying acute severity and determining acute treatment response in patients with schizophrenia.

Methods

We retrospectively examined 251 inpatients diagnosed with schizophrenia and classified them according to treatment (intravenous haloperidol vs. oral antipsychotic medication during the acute phase) and investigated their NLR and NAR while receiving inpatient care.

Results

A total of 48 inpatients were given intravenous haloperidol to manage their acute symptoms; 208 were given oral antipsychotics. The intravenous haloperidol group experienced more severe symptoms, such as agitation and disorganized thinking, during the acute phase. Further, those who received intravenous haloperidol had significantly higher Clinical Global Impression-Severity (CGI-S) scores than the oral antipsychotic group. NLR and NAR were also significantly higher in the haloperidol intravenous group.

Conclusion

Elevated NLR and NAR could be easily measured in patients with psychomotor agitation who should be treated at any facility. Further, they are useful biomarkers for determining disease severity and the effects of treatment on psychomotor excitement in patients who require intravenous haloperidol.

## Introduction

Schizophrenia symptoms are described as positive, such as hallucinations and delusions, and negative, like decreased emotional expression, lack of motivation, and cognitive decline. Over time, schizophrenia may interfere with an individual's social life, increase the likelihood of unemployment, and decrease life expectancy by 10-20 years. Patients who have poor adherence to their medication regimen are more likely to discontinue and tend to relapse repeatedly. Although investigations into the causes of schizophrenia have been conducted over several decades, the condition remains poorly understood. Genetic involvement is suspected because the incidence of schizophrenia in identical twins is approximately 50% and the incidence of schizophrenia in the offspring is approximately 10 times higher when both parents have schizophrenia. Although various theories regarding the causes of schizophrenia have been reported, they have not yet led to direct clinical application [1].

The prevalence of schizophrenia is approximately 1%; however, environmental factors are thought to play a role in the onset of the disease. For example, the incidence of schizophrenia is higher in individuals who reside at high latitudes or in densely populated urban areas [2]. Patients with schizophrenia also present with various symptoms, including positive and negative symptoms. Therefore, in terms of treatment, it is difficult to objectively assess the severity of symptoms and determine the appropriate treatment options.

In cases of severe psychomotor agitation, it is important to determine the appropriate treatment method, such as the intravenous (IV) administration of antipsychotic drugs and modified electroconvulsive therapy. Therefore, in the acute treatment of schizophrenia, prompt and appropriate management strategies should be selected, and biomarkers must be used to objectively determine the severity of symptoms and to predict treatment outcomes.

One etiological theory of schizophrenia suggests a relationship with neuroinflammation. Neuroinflammation involves the activation of microglial cells and increased peripheral benzodiazepine receptor expression. Postmortem studies have reported that schizophrenia is associated with increased numbers of activated microglial cells. Recent studies measuring peripheral benzodiazepine receptors using positron emission tomography (PET) scans have also reported that neuroinflammation is an important factor affecting the onset of schizophrenia symptoms [3]. Inflammation may increase the permeability of the blood-brain barrier (BBB), permitting localized central nervous system lesions to spread to the periphery and allowing infectious pathogens to invade, further damaging the central nervous system [4].

Brain imaging tests are used to evaluate central nervous system functioning. However, such tests are challenging for acutely agitated patients to complete. Moreover, some psychiatric treatment facilities lack brain imaging equipment. Importantly, most treatment facilities have the necessary equipment to carry out blood tests to determine biomarkers. Among the various blood tests that could be useful, tests that examine immunological and inflammatory mechanisms may help examine patients with schizophrenia. For example, previous studies have examined changes in white blood cells (WBCs), particularly lymphocytes; however, no consensus has been reached.

The neutrophil-lymphocyte ratio (NLR) has gained recent attention as a new biomarker for many diseases. NLR comprises a simple ratio of neutrophil-to-lymphocyte counts obtained from peripheral blood. NLR is a biomarker that links two aspects of the immune system: the innate immune response by neutrophils and adaptive immunity by lymphocytes. Furthermore, NLR is a prognostic predictor known to correlate independently with mortality in various diseases such as sepsis, coronavirus disease 2019 (COVID-19), and cancer [4]. The neutrophil-albumin ratio (NAR), platelet-lymphocyte ratio (PLR), and C-reactive protein (CRP)-albumin ratio (CAR) are other promising biomarkers for use in patients with cancer, sepsis, and heart failure [5-7].

Individuals with schizophrenia commonly have higher NLRs than healthy individuals [8]. Furthermore, they present with low lymphocyte counts and high neutrophil counts, indicating an imbalance in leukocyte distribution. Lymphocyte depletion is observed in inflammatory conditions due to the increased apoptosis of lymphocytes [9]. Therefore, these findings may contribute to our understanding of the inflammatory mechanisms associated with schizophrenia. Furthermore, the use of antipsychotic medications will not affect the NLR [10]. Past studies found that the NLR did not decrease in schizophrenic patients who were treatment-resistant but did increase among patients with schizophrenia who were treatment-responsive. These findings suggest that treatment may have an effect on schizophrenia symptoms [11-13]. NLR has also been shown to be significantly correlated with the Positive and Negative Syndrome Scale (PANSS) and Clinical Global Impression-Severity (CGI-S) scores, as well as aggression, clinical symptoms, and disease severity [14,15].

We hypothesized that inpatients with higher psychomotor agitation would have a higher NLR and examined the NLR as a biomarker for determining acute severity and selecting acute treatments for patients with schizophrenia. We compared patients admitted for acute treatment of schizophrenia according to whether or not they were treated with IV haloperidol, used for severe symptoms, as a biomarker for assessing severity and determining the appropriate treatment. We retrospectively studied the medical records of patients with acute schizophrenia who required hospitalization and who had severe psychomotor agitation, refused oral medication, and required IV haloperidol treatment.

## Materials and methods

Participants

The participants were selected from patients admitted to the psychiatric emergency unit of Showa University Northern Yokohama Hospital in Kanagawa, Japan, between January 2014 and December 2019. This hospital provides acute psychiatric inpatient treatment. All patients with schizophrenia were diagnosed according to the criteria of the Diagnostic and Statistical Manual of Mental Disorders, 5th Edition (DSM-5) [16]. Patients younger than 16 years were excluded because it is difficult to confirm the diagnosis and adjust the appropriate dosage of antipsychotics in children and adolescents. Patients who were not treated for a physical disease that can significantly affect blood counts were recruited. However, patients who died or were discharged and those with inflammatory diseases, such as infections, metabolic diseases, advanced-stage cancer, trauma, and collagen diseases, and blood hematopoietic diseases, such as leukemia, were excluded to prevent the effect of changes in WBC count and CRP levels.

We retrospectively analyzed clinical data from the patients' electronic medical records. Following data extraction, we categorized the participants into two groups based on their antipsychotic treatment: those who required IV haloperidol upon admission and those who were administered oral antipsychotic drugs.

Patients were prescribed IV haloperidol if they refused oral antipsychotic drugs or demonstrated extremely severe symptoms, including (1) an imminent risk of suicide attempts or self-harm; (2) pronounced hyperactivity or restlessness; and/or (3) the patient's condition, without treatment, is life-threatening.

Clinical data

We analyzed various demographic and social factors, including age, sex, education, and marital and cohabitation status. We additionally gathered clinical data about the duration of the illness, the number of previous hospitalizations, CGI-S score at admission, acute psychiatric symptoms, chlorpromazine equivalent dose, and blood test results at admission.

To evaluate the patient's psychiatric symptoms and condition, we used the criteria outlined in the hospitalization form prescribed in the Act on Mental Health and Welfare for Persons with Mental Disorders or Disabilities. The evaluation items included auditory hallucinations, visual hallucinations, delusions, association loosening, disorganized thinking, flat affect, depressed mood, restlessness, increased irritability, agitation, and stupor.

This was a retrospective study. Hence, it had some limitations. For example, the possibility of psychotropic medication bias, such as the use of other antipsychotics, mood stabilizers, and anxiolytics, and the presence of missing data on symptom rating scale scores and blood test results could not be ruled out.

Blood cell indexes

The following blood test results were analyzed: CRP and blood cell count (including WBC count, platelet count, neutrophil count, lymphocyte count, monocyte count, eosinophil count, and basophil count). We also calculated the NLR (neutrophil count/lymphocyte count), NAR (neutrophil count/albumin (g/dL)), CAR (CRP (mg/dL)/albumin (g/dL)), and PLR (platelet count/albumin (g/dL)) from the same blood test results.

Statistical analysis

All data analyses were performed using IBM SPSS Statistics for Windows, Version 28.0 (Released 2021; IBM Corp., Armonk, New York, United States). The Shapiro-Wilk test was used to check the normality of the study variables' distributions. Pearson's chi-squared test was used to compare categorical variables. Student's t-test was used to compare normally distributed variables, while the Mann-Whitney U test was used for non-normally distributed variables. Mean±standard deviation (SD) and the number of variables were used to present data. Categorical variables are presented as numbers and percentages. Statistical significance was set at p<0.05.

Ethical compliance

This research was carried out using inpatient medical records depicting typical treatment protocols. We used deidentified IDs and computers disconnected from external networks to ensure patient confidentiality. All identifiable data was strictly concealed. Instead of being exempted from the requirement for informed consent, we provided an "opt-out" option on our website. This informed users that their medical data would be used for research purposes and allowed them to opt out. Patients were also given the option to consent or opt out with assurances that their decision would not affect the clinical care they received. The study was designed according to the principles of the Declaration of Helsinki and was approved by the Institutional Review Board of Showa University Northern Yokohama Hospital (approval number: 19H042).

## Results

We enrolled 262 inpatients diagnosed with schizophrenia, out of which 10 were excluded due to complications of infection and one was excluded due to death. Therefore, we analyzed 251 patients. Out of these patients, 102 were males and 139 were females. The mean±SD for NLR was 3.27±2.91, and for NAR, it was 1099.17±546.

**Figure 1:**
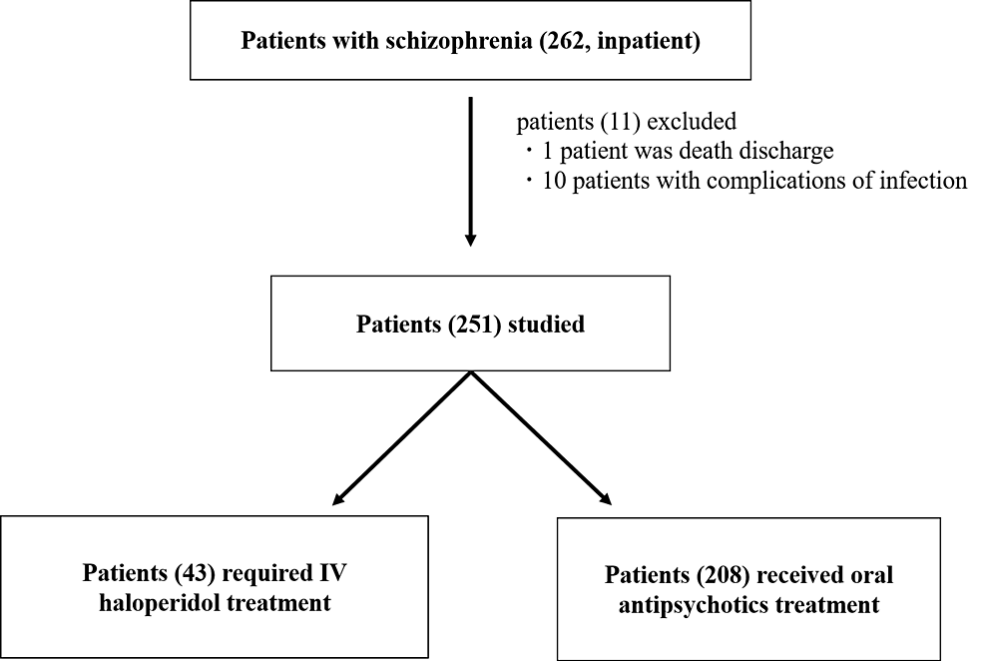
Classification of participants Overall, 262 patients with schizophrenia requiring hospitalization were selected. Overall, one death and 10 patients with infectious complications were excluded. Hence, 251 patients were included in this study. Patients were divided into two groups: those who required IV haloperidol treatment and those who received oral antipsychotic treatment.

The study had 43 patients in the IV haloperidol group and 208 patients in the oral antipsychotic group (Figure 1). Following the post-hoc analysis, we rejected the null hypothesis of the population means of these two groups being equal, with a probability (power) of 0.845.

Demographic characteristics and background information of the participants

The study cohort consisted of two groups: an IV haloperidol (n=43; 17 males and 26 females) with a mean age of 42.65±13.12 years and an oral antipsychotic group (n=208; 85 males and 123 females) with a mean age of 41.34±13.83 years (Table 1). The groups were similar in age and sex and other characteristics such as marital status, smoking habits, family history, and living situation.

**Table 1:** Demographic and clinical data for the IV haloperidol (n=43) and oral antipsychotic groups (n=208) Statistical analysis was performed using the Mann-Whitney U test (a) and chi-squared test (b). NA: not applicable; SD: standard deviation; CGI-S: Clinical Global Impression-Severity

Characteristic	IV haloperidol (n=43)	Oral antipsychotics (n=208)	p-value
Age^a ^	42.65±13.12	41.34±13.83	0.57
Sex^b^: male; female	17; 26	85; 123	0.87
Solitary life^b^	14	43	0.09
Married^b^	13	63	0.947
Divorced or widowed^b^	4	25	0.452
Family history^b^	15	75	0.700
Smoking habits^b^	7	31	0.452
Duration of education^a^	13±2.06	12.6±2.45	0.348
Duration of illness^a^	15.95±13.31	14.72±10.38	0.839
Number of hospitalizations^a^	2.67±4.15	2.24±2.39	0.948
Chlorpromazine equivalent^a^	384.51±462.52	573.67±657.9	p<0.05
CGI-S	5.56±1.13	5.09±1.06	p<0.05
Haloperidol total mg (IV)^a^	25.33±23.38	NA	NA
Haloperidol infusion duration (days)^a^	2.14±1.75 (median: 1)	NA	NA

However, the IV haloperidol group showed significantly worse psychiatric symptoms at admission than the oral antipsychotic group, as indicated by the CGI-S score (p<0.05, as shown in Table 1). Additionally, the chlorpromazine equivalent dose was significantly lower in the IV haloperidol group than in the oral antipsychotic group (p<0.05, as shown in Table 1).

Acute psychosomatic symptoms

There were significant between-group differences for the following symptoms: delusions (53.49% IV vs. 72.6% oral), disorganized thinking (32.56% IV vs. 12.98% oral), depressed mood (0% IV vs. 7.69% oral), restlessness (0% IV vs. 11.06% oral), and agitation (25.58% IV vs. 11.06% oral) (Table 2). The differences were statistically significant (p<0.05) for delusions, depressed mood, restlessness, and agitation and highly significant (p<0.01) for disorganized thinking (Table 2). However, there were no significant between-group differences for auditory hallucinations, visual hallucinations, association loosening, flat affect, increased irritability, and stupor (Table 2).

**Table 2:** Psychiatric symptoms in the IV haloperidol and oral antipsychotic groups Statistical analysis was performed using the chi-squared test.

	IV haloperidol (n=43)	Oral antipsychotics (n=208)	p-value
	% (n)	% (n)	
Auditory hallucinations	39.53 (17)	53.85 (112)	0.09
Visual hallucinations	2.33 (1)	4.33 (9)	0.47
Delusions	53.49 (23)	72.6 (151)	<0.05
Association loosening	6.98 (3)	14.42 (30)	0.19
Disorganized thinking	32.56 (14)	12.98 (27)	<0.01
Flat affect	6.98 (3)	16.83 (35)	0.10
Depressed mood	0 (0)	7.69 (16)	<0.05
Restlessness	0 (0)	11.06 (23)	<0.05
Increased irritability	20.93 (9)	20.67 (43)	0.97
Agitation	25.58 (11)	11.06 (23)	<0.05
Stupor	9.3 (4)	3.85 (8)	0.13

Comparison of the blood cell indexes

There were significant differences between the IV haloperidol and oral antipsychotics for WBC count (8175.35±3441.82 IV vs. 6680.58±2566.97 oral, Mann-Whitney U test, p<0.01) and neutrophil count (5992.96±2960.96 IV vs. 4337.54±2221.31 oral, Mann-Whitney U test, p<0.01) (Table 3).

**Table 3:** Between-group comparison of laboratory variables of interest Statistical analysis was performed using the Mann-Whitney U test. WBC: white blood cell; CRP: C-reactive protein; NLR: neutrophil-lymphocyte ratio; NAR: neutrophil-albumin ratio; CAR: CRP-albumin ratio; PLR: platelet-lymphocyte ratio; SD: standard deviation

	IV haloperidol (n=43)	Oral antipsychotics (n=208)	p-value
	Mean±SD	Mean±SD	
WBC count	8175.35±3441.82	6680.58±2566.97	<0.01
Neutrophil count	5992.96±2960.96	4337.54±2221.31	<0.01
Lymphocyte count	1839.53±782.08	1707.64±886.03	0.27
Albumin (g/dL)	4.31±0.69	4.23±0.59	0.61
CRP (mg/dL)	0.77±1.06	0.64±1.27	0.08
NLR	4.03±3.55	3.11±2.89	<0.05
NAR	1383.07±698.64	1037.67±546	<0.05
CAR	0.2±0.31	0.17±0.33	0.11
PLR	5.82±1.68	5.79±1.97	0.82

The group that received IV haloperidol had a significantly higher NLR (4.03±3.55 vs. 3.11±2.89, Mann-Whitney U test, p<0.05) and NAR (1383.07±698.64 vs. 1037.67±546, Mann-Whitney U test, p<0.05) than those who received oral antipsychotics. However, the two groups did not significantly differ in terms of lymphocyte, CRP, CAR, or PLR levels (Table 3). Moreover, the group that received IV haloperidol had significantly higher WBC and neutrophil counts than the group that received oral antipsychotics. Lymphocyte counts and albumin levels did not differ between the two groups. Therefore, the significant difference between NLR and NAR could be attributed to the neutrophil counts.

## Discussion

This study examined potential biomarkers of imminent psychomotor arousal in patients with schizophrenia. Patients who refused oral antipsychotic drugs were offered IV haloperidol treatment. IV haloperidol was also used with patients who demonstrated extremely severe symptoms, including imminent suicide attempts or self-injurious behavior, hyperactivity, or restlessness, particularly if nontreatment would be life-threatening. In the IV haloperidol group, symptoms such as disorganized thinking and agitation were pronounced, and the CGI-S score was higher than that in the oral antipsychotic group at the time of admission. Similarly, NLR and NAR were higher in the IV haloperidol group than in the oral antipsychotic group. These patients demonstrated psychomotor agitation so imminent that they did not fully understand the need for antipsychotic treatment and, therefore, were administered IV haloperidol.

We considered blood cell indexes as biomarkers of schizophrenia severity and treatment selection because such tests are easily performed in inpatient facilities. NLR and NAR can be calculated using only biochemistry and blood counts, commonly performed in blood tests and attracting attention as simple, inexpensive markers that can be tested at any facility [4].

Previous studies have suggested that NLR is higher in patients with schizophrenia than in healthy controls [8,15]. In some meta-analyses of NLR in schizophrenia, patients' NLR values were 2.63, and healthy controls' NLR values were 1.78 [17]. Patients' mean NLR was 2.03-3.24, whereas healthy controls had a mean NLR range of 1.6-2 [18]. Our study's mean of patients' NLR was 3.28±2.9. Compared with previous studies, this study's participants were considered typical patients with schizophrenia.

Previous studies reported on relationships between NLR and schizophrenia symptom severity. In a study of 22 patients with schizophrenia, NLR was significantly correlated with PANSS-total, PANSS-positive, PANSS-general, and CGI scores and was reduced after long-term antipsychotic treatment. Increased NLR is associated with severe schizophrenia symptoms [14]. Labonté et al. found that patients with schizophrenia who were treatment-resistant showed no decrease in NLR; however, NLR did decrease following treatment among treatment-responsive patients with schizophrenia. NLR may, therefore, be useful for determining treatment response and assessing a patient's symptoms [12]. A positive correlation between aggression and NLR has also been reported. Patients with schizophrenia who are highly aggressive have higher pretreatment NLR than their nonaggressive counterparts. Therefore, NLR could be used as a biomarker to assess aggressive behavior [15]. Kulaksizoglu and Kulaksizoglu showed significant correlations between PANSS-total and NLR, indicating that NLR is related not only to the pathophysiology of schizophrenia but also to clinical symptoms [19].

Patients who required IV haloperidol treatment due to their psychomotor agitation had higher NLR values, CGI-S scores, and rates of symptoms such as perplexing thoughts and agitation. These findings are consistent with previous studies that showed a relationship between NLR and severity. Additionally, the study revealed new insights into the types of symptoms that are severe enough to require IV haloperidol treatment.

NAR reflects hyperinflammation and is used to predict pathologic complete remission in patients with pancreatic and rectal cancer [6,20,21]. A previous study found that patients' NAR was higher compared to that of healthy controls; the authors considered NAR useful in diagnosing schizophrenia [21]. However, there are few studies of NAR for psychiatric disorders. The mean of NAR in our participants was higher in the IV haloperidol group than in the oral antipsychotic group. Higher values for NAR and NLR appear to be associated with more severe psychiatric symptoms. Therefore, evaluating NLR and NAR from blood tests and seeing how the values fluctuate relative to psychotic symptom severity may help physicians with treatment planning. NLR values may be useful in determining the need for invasive therapeutic intervention when oral treatment is unavailable.

Of course, our results should be considered alongside several study limitations. Firstly this was a single-center study. Our cohort was from the psychiatric emergency unit of a general hospital. Hence, it is an incomplete representation of the general population. Moreover, there was a bias toward patients with schizophrenia who had chronic illnesses, and the area had only a few elderly patients with schizophrenia. Secondly, our data were collected retrospectively. Therefore, we could not exclude the possibility of psychotropic medication bias, including the use of other antipsychotics, mood stabilizers, and anxiolytic drugs. Furthermore, some important data could have been missing. Because the data were collected a long time back, there was a possibility of heterogeneity in terms of patient information, such as changes in the tests and treatments during that period of time. Thirdly, some values needed to be added to the symptom assessment. Importantly, only the CGI-S was administered to patients to assess psychiatric symptoms on admission and not after that. Therefore, no pre-to-post comparisons were carried out. The Brief Psychiatric Rating Scale and PANSS were not measured in all patients and were not available to assess schizophrenia symptoms. Lastly, we only assessed NLR and NAR at admission because the timing of blood tests performed after hospitalization was irregular. Moreover, we failed to analyze changes in NLR and NAR after symptom improvement and did not attempt to correlate NLR and NAR levels with changes in symptom severity. This study did not examine patients with schizophrenia who were aged <16 years. Recently, previous studies have reported that patients with schizophrenia who were aged <18 years have a higher NLR than healthy controls and adults [22,23]. Therefore, a study design that excludes age limitations should be considered.

To tackle the issues at hand, additional research studies that examine the psychiatric symptoms of schizophrenia are needed. Such studies should include the results of blood tests taken at admission, during treatment, and at discharge. Furthermore, the research should analyze the correlation between changes in NLR and NAR and the changes in symptom severity.

Our study found that patients with higher levels of psychomotor arousal had higher NLR and NAR values. This implies that blood tests may be useful for objectively assessing the level of psychomotor arousal during psychiatric exacerbations. However, we recommend validating these findings within the context of adequately powered, prospective studies.

## Conclusions

Herein, patients with severe psychosomatic schizophrenia who could not receive oral antipsychotics and required hospitalization for IV haloperidol had higher CGI-S and increased NLR and NAR compared with patients who could receive oral treatment. In the IV haloperidol group, patients also had higher rates of psychiatric symptoms such as delusions and agitation compared with patients who could be orally treated.

In clinical practice, there are many situations in which the lack of objective biomarkers of psychiatric symptoms makes it difficult to decide whether or not to provide IV antipsychotic treatment. In such cases, elevated NLR and NAR were considered useful for IV haloperidol treatment selection as biomarkers that could be easily measured in patients with psychomotor agitation who should be treated.

This study and previous reports showed that NLR and NAR could be an objective indicator that is useful for disease diagnosis, severity determination, and treatment selection in patients with schizophrenia. Nevertheless, larger, multicenter, prospective studies should be performed to validate our results.
